# Supplementary material for: Fecal biomarkers of environmental enteric dysfunction and the gut microbiota of rural Malawian children: An observational study
Source: Heliyon. 2021 Oct 16;7(10):e08194. doi: 10.1016/j.heliyon.2021.e08194 (PMC8554169; doi:10.1016/j.heliyon.2021.e08194)
Supplement: Supplementary table [file mmc2.docx]

**Article title: Fecal biomarkers of environmental enteric dysfunction and the gut microbiota of rural Malawian children: an observational study**

**Supplementary table**

**S1: Metabolic pathways predicted by PICRUSt2 analysis**

| **Metabolic pathway** | **Count score sum for each predicted pathway** |
| --- | --- |
| Proteinogenic Amino Acid Biosynthesis | 15784472 |
| Purine Nucleotide Biosynthesis | 9236611 |
| Vitamin Biosynthesis | 7194008 |
| Sugar Biosynthesis | 5100685 |
| Cell Wall Biosynthesis | 4291038 |
| Fatty Acid Biosynthesis | 4143453 |
| Phospholipid Biosynthesis | 4110331 |
| Pyrimidine Nucleotide Biosynthesis | 3588915 |
| 2 Deoxyribonucleotide Biosynthesis | 3315321 |
| Sugar Degradation | 2854327 |
| Polysaccharide Degradation | 1969759 |
| Terpenoid Biosynthesis | 1875327 |
| Purine Nucleotide Degradation | 1836367 |
| Fermentation to Acetate | 1602071 |
| Chorismate Biosynthesis | 1595960 |
| Quinol and Quinone Biosynthesis | 1354676 |
| Metabolic Clusters | 1349440 |
| NAD Metabolism | 1224545 |
| Polyprenyl Biosynthesis | 1204621 |
| Coenzyme A Biosynthesis | 1136914 |
| Sugar Derivative Degradation | 932614 |
| Glycan Biosynthesis | 850382 |
| Sugar Acid Degradation | 804311 |
| CO_2_ Fixation | 597805 |
| Other Amino Acid Biosynthesis | 583747 |
| Lipopolysaccharide Biosynthesis | 569489 |
| Tetrapyrrole Biosynthesis | 496428 |
| Pyrimidine Nucleotide Degradation | 447537 |
| Sulfur Compound Metabolism | 384679 |
| Porphyrin Compound Biosynthesis | 327754 |
| Proteinogenic Amino Acid Degradation | 260546 |
| Nitrogen Compound Metabolism | 243591 |
| 8 Amino 7 oxononanoate Biosynthesis | 213254 |
| Formaldehyde Assimilation | 88494 |
| Phenolic Compound Degradation | 64477 |
| Generation of Precursor Metabolite and Energy | 61787 |
| Siderophore Biosynthesis | 52992 |
| Formaldehyde Oxidation | 48703 |
| 4 Aminobutanoate Degradation | 44988 |
| Phosphorus Compound Metabolism | 44779 |
| Fatty Acid Degradation | 42828 |
| Toluene Degradation | 40837 |
| Allantoin Degradation | 38424 |
| Propanoate Degradation | 29836 |
| Glycerol Degradation | 10073 |
| Catechol Degradation | 6892 |
| Coenzyme B | 2076 |
| Coenzyme M Biosynthesis | 1670 |
| Coenzyme F420 | 1591 |
| Reductant Biosynthesis | 1197 |
| Protocatechuate Degradation | 507 |
| Gallate Degradation | 194 |

*The pathways have been ranked from those with the highest count score sum to the lowest.*
